# Exploring the influencing factors of quality of life among the empty nesters in Shanxi, China: a structural equation model

**DOI:** 10.1186/s12955-021-01793-x

**Published:** 2021-05-28

**Authors:** Chichen Zhang, Yuan Cai, Yaqing Xue, Xiao Zheng, Xiaozhao Yang, Jiao Lu, Lihong Hou, Mimi Li

**Affiliations:** 1 School of Health Management, Southern Medical University, No. 1023, South Shatai Road, Guangzhou, 510515 Guangdong China; 2 Department of Health Management, Nanfang Hospital, Southern Medical University, Guangzhou, Guangdong China; 3 School of Management, Shanxi Medical University, Taiyuan, Shanxi China; 4 School of Public Health, Southern Medical University, Guangzhou, Guangdong China; 5 School of Sociology and Anthropology Sun Yat-Sen University, Guangzhou, Guangdong China; 6 The Second Affiliated Hospital, Shaanxi University of Chinese Medicine, Xi’an, Shaanxi, China; 7 Jincheng People’s Hospital, Jincheng, Shanxi China

**Keywords:** Quality of life, Influencing factors, Empty nesters

## Abstract

**Background:**

As China’s aging trend intensifies and the proportion of empty nests increases, the health-related quality of life of the elderly is the focus of social attention. Previous studies focused on the quality of life of the elderly, rather than empty nesters, and lacked the discussion of the mechanism of influencing factors. Thus, this study aimed to explore the influencing factors of the quality of life (QOL) and interaction mechanisms among empty nesters in Shanxi Province, China, so as to provide practical reference for improving the QOL of empty nesters.

**Methods:**

A total of 4901 empty nesters in Shanxi Province, China, were selected using multi-stage random cluster sampling method in this cross-sectional study. The quality of life was assessed with the Short Form 36 Health Survey (SF-36). Multiple linear regression analysis (stepwise) was performed to examine the factor associated with QOL. Structural equation model (SEM) approach was conducted to test the direct and indirect association between QOL influencing factors with QOL.

**Results:**

The average QOL score of empty nesters was 63.34 (SD = 17.23). The multiple linear regression revealed that gender, age, education, monthly income, drinking status, exercise frequency, physical examination frequency, attention to daily nutritional health, relationship with a spouse and relationship with children were significant predictors of the empty nester’s QOL (*P* < 0.05) (*R*^2^ = 0.128). SEM showed that behavioral lifestyle had a direct effect on QOL (*β* = 0.446, *P* < 0.001). Socio-economic status had an indirect effect (*β* = 0.288, *P* < 0.001) on QOL through behavioral lifestyle. The family relationship had an indirect effect (*β* = 0.115, *P* < 0.001) on QOL.

**Conclusion:**

Behavioral lifestyle was the strongest influencing factor in the quality of life among empty nesters, followed by socio-economic status and family relationships. Thus, maintaining a healthy behavioral lifestyle was important to improve the QOL of the empty nesters. Our findings provide a concrete and strong reference for the formulation of targeted intervention strategies.

## Background

In the twenty-first century, with society’s progress and the development of the science and technology, the world is undergoing profound changes. Changes in the age structure of the population indicate that the aging process gradually speeds up. According to the annual data of the National Bureau of Statistics of China (CNBS), the Chinese aged 60 years and above accounted for 18.1% of the total population in 2019, reaching 178 million [1]. The unprecedented elderly population has brought a series of problems and challenges to China’s social and economic development, among which the empty nest elderly is more worthy of attention [2]. The ‘empty nester’ refers to the elderly (age ≥ 60 years old) who live alone or with their spouse, because they have no children or their children are married or work outside for a long time [3]. Due to new socioeconomic changes, including the imbalance of regional economic development and the rapid development of urbanization in China, many youth rural laborers have flowed to developed regions, leaving the elderly at home [4]. Furthermore, affected by the one-child policy, the ‘4–2–1’ (four grandparents, two parents and one child) single-family structure emerged in China. Some elderly live alone or only with their spouses [5]. With the transformation of the family life cycle, and the trend of independent living for children after marriage, the traditional family system’s position is weakened, the ‘empty nest’ phenomenon is becoming increasingly significant, and an inevitable trend in China [6, 7]. The empty nest situation will increase the time spent by the elderly in agriculture and family labor in rural areas, and the prevalence of chronic diseases among empty nesters in rural areas is higher [8]. The financial support and spiritual consolation of the elderly face uncertainty in an empty nest situation [9]. Thus, paying attention to the empty nester is, therefore, an essential component of public health.

Recently, interest in the quality of life (QOL) of empty nesters has gained momentum. The QOL is a multi-dimensional construct that includes three aspects of physical, mental and social functioning [10]. With the increase of age, the elderly often faces the problem of physiological function decline, which has a high incidence of chronic diseases. A cross-sectional study in Belgium showed that the prevalence of multimorbidity was 82.6% in the elderly aged 65 and above [11]. These irresistible physiological declines will bring physical pain and economic burden to the elderly [12]. In addition, affected by the traditional concept of ‘bring up their children for old age’, the elderly are more inclined to receive the care and attention of their children [13]. Yet, with the gradual shrinking of the family size and the disintegration of the traditional extended family structure, the function of family care for the elderly has gradually weakened, and the elderly cannot receive direct life care, which reduces their quality of life indirectly. Previous studies have showed that an empty nest has a negative impact on the health of the elderly. A cross-section study from China showed that empty nest elders had worse self-care ability and lower mental health scores [14]. Depression, anxiety, loneliness and other mental illnesses are common among empty nesters, and these mental illnesses seriously affect their quality of life [15]. Zhang et al. found that 64.2% of empty nesters have depression [16], and 44.5% of empty nesters reported anxiety [17]. Mental health problems loom large for the empty nesters. Mental disorders not only reduce the social activities of the elderly, but also makes them further deteriorate in social functions [18], and reduces their quality of life [19]. Moreover, QOL is an important consideration of health management for older adults, needs public attention.

Many previous studies have evaluated the influencing factors of QOL. Studies revealed that sociodemographic characteristics (age, education level [20], and income level), physical factors (hearing loss [21], chronic disease, smoking [22], lack of physical activities [23]), psychological factors (loneliness [24] and depression [16]), social support [25] factors affect the QOL of the elderly. These studies have undoubtedly advanced our knowledge of the factors affecting the QOL of the elderly. However, these studies did not explore the mechanism of action between influencing factors and QOL, the underlying mechanism remained unclear. More specifically, these studies did not assess the direction and structure of the associations between influencing factors and QOL. Among various influencing factors of the older people’s QOL, socio-economic status has attracted scholars’ extensive attention. The difference in social and economic status leads to inequality in the health of the elderly [26]. Wang’s research revealed that socio-economic status has a positive predictive effect on the health of the elderly. However, its direct explanatory power is not strong, and indirectly affects the health of the elderly through physical exercise, diet and other behavioral lifestyles [27]. The World Health Organization pointed out that 60% of the quality of an individual’s health and life depends on his/her behavior and lifestyle. And studies have revealed that healthy behaviors and lifestyles have a positive predictive effect on the quality of life [28]. A harmonious family relationship helps to improve the quality of life [29]. Based on the above, the present study aims to explore the influencing factors of QOL and the interaction mechanism of various factors. We assumed that there was a significant relationship between socio-economic status, behavioral lifestyle, family relationship and QOL among the empty nesters, while exploring the underlying mechanism between them.

## Methods

### Study design and participants

This study was conducted in Shanxi, China, from June 2016 to July 2017, which consists of 11 cities. We employed a multi-stage random cluster sampling method to select participants. According to the order of 11 cities and subordinate districts/counties on the government’s website, each district/county in every city was numbered. Second, a random number table was employed to select two districts /counties in each city, which can draw samples randomly and equably. Third, two communities/administrative villages were randomly chosen from each selected district/county in the same way. Fourth, the same method was used to extract two residential districts/natural villages from each community/administrative village. All empty-nesters in selected residential districts/natural villages who met the criteria were enrolled in this study. Elders aged 60 years and above, had no children, living alone or with a spouse, could communicate in Chinese, and with no cognitive disorders, were eligible for the study. Elders were excluded when they were diagnosed with cognitive disorders or serious diseases. The sample size was estimated according to the following formula $N= [{U}_{\alpha } S/\delta ]{ }^{2}$. Previous research showed that the difference between the standard deviation of the PCS and MCS scores in SF-36 is about 3 [22, 30]. So, the *S* was determined to 3 in this study. If *U*_*α*_ = 1.96, *S* = 3, *δ* = 0.1, the estimated sample would be 3458 participants. Considering the 20% loss to follow-up rate and random errors, the final sample size was estimated to be 4335 at least. Factually, 5000 participants were investigated. We interviewed 5,000 people who met the criteria, and 4,901 completed the questionnaire. The effective response rate was 98.02%.

All participants were informed of the research’s purpose and procedure upon their recruitment and obtained their consent. For the participants who were in poor health or had limited communication abilities, consent was obtained from their children or legal guardians. All study procedures were approved by the Ethics Committee of Shanxi Medical University.

### Social demographic characteristics

Social demographic characteristics included gender, age, residence, education level, marital status, monthly income. The demographic characteristics were categorized as follows: gender (male vs. female), age (60–69, 70–79 and 80 years or above), residence (urban vs. rural), education level (no formal education, primary school, secondary school, high school, junior college and university or higher), marital status (married vs. single), monthly income (none, < 1000RMB, 1000–3000 RMB and > 3000 RMB).

### Behavioral lifestyle

Behavioral lifestyle included smoking, drinking, exercise frequency, physical examination frequency and attention to daily healthy diet were collected. Exercise frequency was categorized as none, 1–2 times per week, 3–5 times per week, 6 or more times per week. Physical examination frequency was categorized as never, irregular, regular physical examination every few years, annual physical examinations. Daily diet health was measured by a question of “Do you pay attention to the daily healthy diet?”. The answer included “No”, “Less”, “More” and “Most”.

### Social activity participation

The degree of participation in social activities was measured by a question of “What do you think about your participation in social activities?” The answer is “none and low/moderate/high”.

### Family relationship

Self-reported relationship with spouse and self-reported relationship with children were categorized as bad and close.

### The 36-item short-form health survey (SF-36)

This study applied the Chinese version of the SF-36, which consists of 36 items to evaluate the quality of life (QOL) of participants [31]. One item assesses the perceived change in health status, and the remaining 35 items assess the physical and mental components of health [32]. The SF-36 included 8 dimensions: physical functioning (PF), role-physical (RP); bodily pain (BP); general health (GH); vitality (VT); social functioning (SF); role-emotional (RE) and mental health (MH). These 8 dimensions are summarized in 2 categories that physical component summary (PCS) and mental component summary (MCS). PCS includes PF, RP, BP and GH. MCS includes VT, SF, RE, and MH. Each dimension score was converted to a range from 0 to 100, with a high score indicating better levels of functioning. The formula to transformed scores is as follows: transformed scores = [(actual raw score − lowest possible raw score)/possible raw score range] × 100 [33]. The SF-36 score was obtained from the average of the 8 dimensions. Consequently, higher SF-36 scores indicate better QOL. Additionally, the Chinese version of the SF-36 has been extensively validated. The Cronbach's α of the SF-36 was 0.834 in this study.

### Statistical analysis

All data analyses were performed by SPSS 22.0 and AMOS 22.0 statistical software. Independent-sample *t*-test and one-way ANOVA were used to compare the demographic differences. Multiple linear regression analysis (stepwise) was used to explore influential factors of QOL. The inclusion criterion sets at a *p*-value smaller than 0.05, and the exclusion criterion was a *p*-value greater than 0.1. The structural equation model was used to quantify the hypothesized association between influencing factors and QOL. Overall model goodness of fit was assessed using *χ*^2^ /df ≤ 5.0, goodness-of-fit index (GFI) ≥ 0.90, adjusted goodness-of-fit index (AGFI) ≥ 0.90, root mean square error of approximation (RMSEA) ≤ 0.08, comparative fit index (CFI) ≥ 0.90, and Tucker-Lewis index (TLI) ≥ 0.90 [34]. Multicollinearity was assessed using the tolerance and variation inflation factor (VIF). For each variable included, the variance inflation factor (VIF) ranged from 1.062 to 1.486, and tolerance ranged from 0.673 to 0.941, suggesting that the absence of multicollinearity in the model.

## Results

A total of 4901 empty nesters were recruited in this study, of whom 2546 (51.9%) were males and 2355 (48.1%) were females. The mean age was 68.5 (SD = 2.5) years, 65.0% of the participants lived in rural areas, 62.7% of empty nesters with low education, 68.2% of empty nesters were married, and 62.1% of the participants had no monthly income or income less than 1,000 RMB. This survey shows that the average quality of life score of the empty nester was 63.34 (SD = 17.23). The quality of life scores for different types of demographics were shown in Table 1. There were statistically significant differences in the QOL for demographic characters of gender, age, residence, education, marital status, monthly income, drinking status, exercise frequency, physical examination frequency, attention the daily healthy diet (*P* < 0.05). There were significant differences in QOL scores among empty nesters with different participation in social activities, relationship with spouse, and relationship with children (*P* < 0.05), there were no significant differences between them in the aspects of smoking status (*P* > 0.05; Table 1).

**Table 1:** Description and analysis of QOL among empty nesters in Shanxi China

Characteristics	N (%)	QOL score (mean ± SD)	*t*/*F*	*P*
Gender				
Male	2546 (51.95)	64.59 ± 17.21	5.291	< 0.001
Female	2355 (48.05)	61.99 ± 17.16		
Age				
60–69	2440 (49.79)	65.34 ± 16.66	51.381	< 0.001
70–79	1910 (38.97)	62.47 ± 17.30		
80–	551 (11.24)	57.51 ± 17.93		
Residence				
Urban	1714 (34.97)	66.26 ± 16.92	8.790	< 0.001
Rural	3187 (65.03)	61.78 ± 17.20		
Education				
No formal education	1500 (30.61)	59.10 ± 17.05	39.872	< 0.001
Primary school	1573 (32.10)	63.12 ± 16.85		
Secondary school	1068 (21.79)	65.67 ± 16.54		
High school	531 (10.83)	68.59 ± 17.44		
Junior college	111 (2.26)	70.75 ± 15.35		
University or higher	118 (2.41)	68.66 ± 17.89		
Marital status				
Married	3341 (68.17)	64.84 ± 17.07	27.066	< 0.001
Single (never married, divorced, widowed)	1560 (31.83)	60.39 ± 16.59		
Monthly income				
None	1676 (34.20)	58.80 ± 17.22	86.308	< 0.001
< 1000 RMB	1371 (27.97)	63.16 ± 16.18		
1000–3000 RMB	1287 (26.26)	66.48 ± 16.61		
> 3000 RMB	567 (11.57)	70.09 ± 17.45		
Smoking status				
Never	3214 (65.58)	62.97 ± 17.25	2.178	0.113
Quit smoking	709 (14.47)	64.11 ± 17.08		
Smoking	978 (19.95)	64.01 ± 17.29		
Drinking status				
Never	3417 (69.72)	62.61 ± 17.40	6.997	< 0.001
Occasionally	1168 (23.83)	64.98 ± 16.66		
Often	206 (4.20)	64.79 ± 16.92		
Every day	110 (2.25)	66.10 ± 17.24		
Exercise frequency				
None	1304 (26.61)	58.67 ± 17.83	92.062	< 0.001
1–2 times per week	1663 (33.93)	61.72 ± 16.21		
3–5 times per week	1208 (24.65)	66.67 ± 16.06		
6 or more times per week	726 (14.81)	69.93 ± 17.22		
Physical examination frequency				
Never	380 (7.75)	62.74 ± 16.81	57.857	< 0.001
Irregular	2256 (46.03)	62.85 ± 16.82		
Regular physical examination every few years	925 (18.88)	69.50 ± 16.85		
Annual physical examination	1340 (27.34)	60.10 ± 17.24		
Attention the daily healthy diet				
No	152 (3.10)	56.02 ± 17.95	44.939	< .0001
Less	1326 (27.06)	59.82 ± 17.39		
More	2547 (51.97)	64.35 ± 16.83		
Most	876 (17.87)	67.02 ± 16.76		
Participation in social activities				
None	730 (14.89)	58.89 ± 19.06	37.517	< 0.001
Low	2309 (47.11)	62.39 ± 17.14		
Moderate	1445 (29.49)	66.18 ± 16.06		
High	417 (8.51)	66.63 ± 16.04		
Relationship with spouse				
Have no spouse or bad	591 (12.06)	57.02 ± 17.02	86.996	< 0.001
Close	4310 (87.94)	64.67 ± 16.89		
Relationship with children				
Have no children or bad	537 (10.95)	54.54 ± 17.19	107.049	< 0.001
Close	4364 (89.05)	65.07 ± 16.72		

The stepwise multiple linear regression model was performed to explore the influencing factors of QOL in empty nesters. Sociodemographic variables with statistically significant differences were used as independent variable X and QOL scores were defined as dependent variable Y. The model showed that the significant accounting of the SF-36 variance ranged from 12.8%. The model revealed that (in descending order of standardized regression coefficients) exercise frequency (*β* = 0.133, *P* < 0.001), monthly income (*β* = 0.107, *P* < 0.001), age (*β* = -0.122, *P* < 0.001), relationship with children (*β* = 0.081, *P* < 0.001), physical examination frequency (*β* = 0.066, *P* < 0.001), attention to daily nutritional health (*β* = 0.060, *P* < 0.001), drinking status (*β* = 0.044, *P* < 0.001), education (*β* = 0.052, *P* < 0.001), relationship with a spouse (*β* = 0.051, *P* < 0.001) and gender (*β* = -0.041, *P* < 0.001) were significant predictors of the empty nester’s QOL. Moreover, age was negatively related to the QOL of empty nesters (Table 2).

**Table 2:** Multiple stepwise linear regression of QOL among empty nesters in Shanxi, China

Independent variables	Unstandardized coefficients (*β*)	Standard error	Standardized coefficients (*β*)	*t*	*P*
(constant)	45.483	1.718		26.467	< 0.001
Exercise frequency	2.264	0.248	0.133	9.112	< 0.001
Monthly income	1.813	0.258	0.107	7.014	< 0.001
Age	− 3.084	0.342	− 0.122	− 9.028	< 0.001
Relationship with children	1.211	0.215	0.081	5.633	< 0.001
Physical examination frequency	1.104	0.242	0.066	4.562	< 0.001
Attention to daily nutritional health	1.393	0.338	0.060	4.119	< 0.001
Drinking status	1.112	0.372	0.044	2.985	0.003
Education	0.735	0.218	0.052	3.379	0.001
Relationship with spouse	0.876	0.248	0.051	3.537	< 0.001
Gender	− 1.405	0.512	− 0.041	− 2.744	0.006

**Fig.1:**
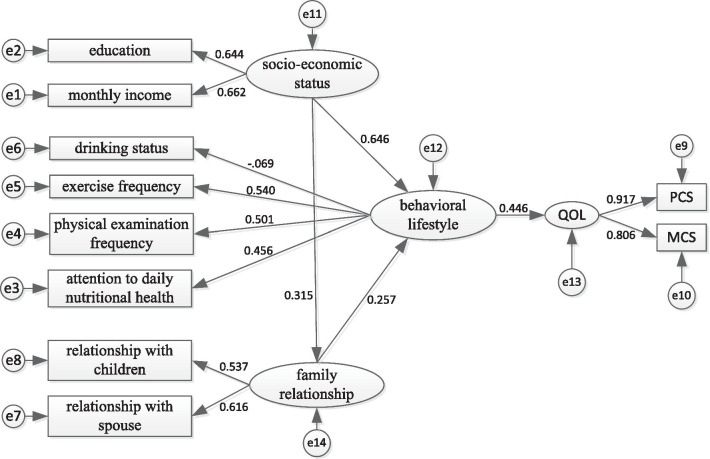
The structural equation model of Quality of Life among empty nesters in Shanxi, China

Structural equation modeling was employed for testing a hypothesized model for QOL among the empty nesters. The hypothesized model consisted of four latent factors (See Fig. 1: socio-economic status, behavioral lifestyle, family relationship, and QOL) and 10 observed variables. Observed variables were statistically significant variables in multiple linear regression. All four latent factors are measured by several observed indicator variables. Analysis of the model of QOL revealed: *χ*^2^ = 217.859, *df* = 29, *χ*^2^/*df* = 7.512 > 5.0, GFI = 0.991 > 0.900, AGFI = 0.983 > 0.900, RMSEA = 0.036 < 0.05, CFI = 0.977 > 0.900, and TLI = 0.965 > 0.900. The assessment suggests that the hypothetical model provides a good fit for the data. However, *χ*^2^ is sensitive to the sample size, due to the large sample size (*n* = 4901) in the study, the *χ*^2^ value was large.

Figure 1 illustrates the model results. Behavioral lifestyle had a direct effect on QOL (*β* = 0.446, *P* < 0.001). Socio-economic status had a direct effect to behavioral lifestyle (*β* = 0.646, *P* < 0.001). Meanwhile, behavioral lifestyle mediated the relationship between socio-economic status and QOL. The indirect effect was 0.288. The family relationship had a direct effect to behavioral lifestyle (*β* = 0.257, *P* < 0.001). The family relationship had an indirect effect on the QOL through behavioral lifestyle, the indirect effect was 0.115. Moreover, the greatest absolute value of standardized total effects was from behavioral lifestyle (0.446), followed by socio-economic status (0.288) and family relationship (0.115). Table 3 presented the total, direct and indirect effects of the variables on QOL.

**Table 3:** Standardized total, direct and indirect effect of the variables on QOL

Path	Total effect	Direct effect	Indirect effect
Socio-economic status → behavioral lifestyle → QOL	0.288	–	0.288
Family relationship → behavioral lifestyle → QOL	0.115	–	0.115
Behavioral lifestyle → QOL	0.446	0.446	–

## Discussion

In the current study, the result showed that the score of QOL was 63.34 (SD = 17.23), which was similar to the results of Jia’ study [35]. The QOL level of the Chinese elderly was quite different from varied survey scenarios. The elderly’s level of QOL under the home care model was high [36], but Li’ study showed that QOL of nursing institutions for the elderly in Shenyang were at a low level [37]. Different regional economic development, measurement instruments and appraisal standards may account for the varied outcome. This study revealed that QOL of empty nesters were significantly influenced by gender, age, education, monthly income, drinking status, exercise frequency, physical examination frequency, attention to daily nutritional health, relationship with children, relationship with spouse. Generally, empty nesters who were the older adults [38], low education level [9] and low-income individuals [39] might have less awareness and ability to maintain health, which means they could not be of better QOL. In addition, we explore the association between socio-economic status, behavioral lifestyle, family relationship and QOL among the empty nesters, and verified the relationship among them. Structural equation model results showed that behavioral lifestyle directly impacted QOL, and socio-economic status and family relationship indirectly affect QOL through behavioral lifestyle. This study provides new insight into the underlying mechanism of the QOL of the empty nesters and its influencing factors. Overall, empty nesters as one of the most vulnerable groups in China, their quality of life needs to be improved.

According to the structural equation modeling, behavioral lifestyle had a direct impact on QOL. And this result was consistent with previous studies, which have shown that differences in behavioral lifestyle such as attention to daily nutritional health [40], drinking status, exercise frequency and physical examination frequency [30] partly explain the worse score of QOL among the elderly. A previous study reported that approximately 50.8% of deaths from some chronic diseases were attributed to an unhealthy lifestyle [41]. However, in China, empty nesters who live independently have no children’s care and low awareness rate of health-related knowledge and lack of professional intervention on behavioral lifestyle. Consequently, they were prone to suffer from chronic diseases or aggravate their condition and reduce the quality of life [42]. The Health Belief Model proposes that individuals perceive the threat of a health risk to be serious and personally susceptible. They are most likely to take preventative action [43]. Based on this, the health management of empty nesters needs to pay more attention to interventions such as individualized health education to improve their self-care awareness and develop healthy behavioral lifestyle habits, thus improving their quality of life.

Another finding of this study was the mediating role of behavioral lifestyle in the relationship between socio-economic status and QOL. The indirect effect of socio-economic status on QOL through behavioral lifestyle was found to be very strong, suggesting that behavioral lifestyle played a critical role in affecting the relationship between socio-economic status and QOL [44]. This result explained the procedure of how socio-economic status affects QOL among the empty nesters in more depth. Groups with higher socioeconomic status had higher incomes and education levels and demanded much more for a healthy lifestyle, which in turn directly improves their QOL. As Gobbens et al. found significant associations between high income and high quality of life in the Dutch elderly after controlling for other variables [45]. Absolute income is robustly associated with health status. Moreover, based on the theory of relative deprivation, relative deprivation income may also partly explain the association between income and population health status [46]. Lower relative income might bring pressure to adversely affect mental health, and worsen the quality of life [47]. A previous study showed that people with higher socioeconomic status were more inclined to physical exercise, and their behavioral lifestyle was healthier and had a better level of health [48]. Consequently, the choice of behavioral lifestyles was related to their economic level. Non-empty nesters living with their children could rely on their children, while empty-nesters did not receive regular and meaningful financial support from children and relatives, incomes are the most direct dependence for empty nesters’ QOL [49]. Higher educational attainment tends to have higher income and socioeconomic status, which tend to better health. However, the elderly with lower education level were prone to misled by negative health information and might not form a healthy lifestyle without professional intervene [50]. Thus, it is recommended to create tailored jobs for empty nesters to ensure economic independence and maintain a healthy life. Encourage the community to advocate healthy behavioral lifestyles, such as through health reeducation to improve the awareness rate of health knowledge among the elderly.

Similarly, this research revealed that family relationship indirectly affects the QOL of empty nesters through behavioral lifestyles. Individuals with strong family support had good QOL compared with others [51]. Previous studies have suggested that close relationship with children was a major factor in improving behavioral lifestyles among the elderly [50]. And the QOL of the elderly with a spouse or living with family was higher than the elderly without a spouse and living alone [52]. Thus, relationships with children and spouses are particularly important to the quality of life of empty nesters. Along with aging, children and spouses are very important emotional sustenance for the elderly. For the Chinese, the family was considered to be closely related to relieving psychological distress through emotional sustenance and is an important source of social support [4]. And a harmonious family relationship means that they have more opportunities for life and emotional communication with their children and spouses, thus getting more help and maintaining a good mental health status [50]. Family support have an important impact on the health of empty nesters. As a study found that elderly who saw their relatives with lower frequency, PSC scores were likely to decrease, prone to depression, loneliness, and other negative emotions [53]. Encourage children or spouse to communicate with empty nesters regularly to relieve negative emotions, and attention should be paid to guide the elderly to choose a healthy lifestyle, thus improving health awareness and quality of life.

This study had some limitations. First, participants in this study were selected from Shanxi Province, thus these results may not be representative of empty nesters in other areas in China. Second, this was a cross-sectional study, the study results failed to determine causal relationships among the variables, so it is crucial to follow up with longitudinal research.

## Conclusion

This study explored the influencing factors of quality of life and the interaction mechanism based on a survey of 4901 empty nesters in Shanxi Province. This study revealed that behavioral lifestyle had a direct effect on QOL among empty nesters. The impact of socio-economic status and family relationships on QOL were mediated by behavioral lifestyle. This research provided scientific evidence for developing effective intervention strategies to improve empty nesters’ QOL. Reinforcing public health services, popularizing health-related knowledge, providing necessary financial support and basic social security is essential to improving the physical and mental health of elderly empty nesters.

## Data Availability

Please contact author for data requests.

## Abbreviations

QOL: Quality of life
SF-36: The 36-item short-form health survey
